# Intra- and interspecific variability among congeneric *Pagellus *otoliths

**DOI:** 10.1038/s41598-021-95814-w

**Published:** 2021-08-11

**Authors:** Claudio D’Iglio, Marco Albano, Sergio Famulari, Serena Savoca, Giuseppe Panarello, Davide Di Paola, Anna Perdichizzi, Paola Rinelli, Giovanni Lanteri, Nunziacarla Spanò, Gioele Capillo

**Affiliations:** 1 Department of Chemical, Biological, Pharmaceutical and Environmental Sciences, University of Messina, Viale F. Stagno d’Alcontres 31, 98166 Messina, Italy; 2 Institute for Marine Biological Resources and Biotechnology (IRBIM), National Research Council (CNR), Section of Messina, Messina, Italy; 3 Department of Veterinary Sciences, University of Messina, Messina, Italy; 4 Department of Biomedical, Dental and Morphological and Functional Imaging, University of Messina, Messina, Italy

**Keywords:** Ecology, Zoology

## Abstract

Otolith features are useful tools for studying taxonomy, ecology, paleontology, and fish biology since they represent a permanent record of life history. Nevertheless, the functional morphology of otoliths remains an open research question that is useful to completely understand their eco-morphology. This study aims to deepen the knowledge of intra- and interspecific variation in *sagitta* morphology in three congeneric seabreams, to understand how such variability could be related to the lifestyles of each species. Therefore, the *sagittae* (n = 161) of 24 *Pagellus bogaraveo*, 24 *Pagellus acarne*, and 37 *Pagellus erythrinus* specimens, collected from the south Tyrrhenian Sea, were analyzed using scanning electron microscopy and a stereomicroscope to assess morphometric features, variability between otolith pairs and the external crystalline structure the of *sulcus acusticus*. Statistical analysis demonstrated that, between the species, variability in sagittal otolith rostral length growth and *sulcus acusticus* features, correlated with increased fish total length and body weight. Moreover, slight differences between otolith pairs were detected in *P. acarne* and *P. erythrinus* (*P* < 0.05). The results confirm changes in otolith morphometry and morphology between different congeneric species and populations of the same species from different habitats.

## Introduction

The inner ear is fundamental for vestibular and acoustic functions (balance and hearing) in teleost fishes. Its structure comprises three semicircular canals and their end organs, the ampullae, and three otolith organs (the *sacculus, utriculus,* and *lagena*). These organs contain three pairs of otoliths (three on each side), known as the *sagitta, lapillus,* and *asteriscus*^1,2^.

The main chemical component of otoliths is calcium carbonate. This is normally in form of aragonite, and other inorganic salts, associated with a protein matrix from which the otoliths develop^3^.

Otoliths are one of the most studied elements of teleost fish anatomy because they represent a permanent record of life history. Due to their species-specific morphology, otoliths are especially important in taxonomy and are a useful tool for distinguishing species among large numbers of bony fishes^1,4–10^. Several factors affect the morphology, morphometry, and microstructure of otoliths. These include environmental factors (e.g., water depth, temperature, salinity, and substrate)^11^, feeding habits^12,13^, ontogeny^14^, physiology (e.g., hearing capabilities associated with acoustic communication)^2,15^ and phylogeny^16^. In recent decades, otolith shape analysis has become fundamental in fisheries management for differentiating between fish stock, populations and their migration^17,18^, and eco-geochemistry^19^.

The *sagitta* (or saccular otolith) is usually the largest otolith and displays the highest inter-specific morphological diversity (exceptions include some otophysan species, in which the utricular otoliths are much larger than the *sagitta*, e.g., *Arius felis* (Linnaeus, 1766)^20^). Therefore, it is the most studied otolith. It is linked to the *macula sacculi* by a depression (called the *sulcus acusticus*) on the mesial face. The *macula sacculi* are indirectly attached to the complete *sulcus acusticus* via the otolithic membrane. The *sulcus acusticus* is composed of two areas, the *ostium* (anterior, generally in the rostral position) and the *cauda* (posterior), which are connected by the *collum*. Morphological features, shape, and the crystalline structure of the *sulcus* are occasionally used to differentiate between different fish stock, species, and size relationships within populations, regarding the environmental, biological, and ecological behavior of the species^21–23^. Conversely, otolith morphology has long been used to distinguish between species, and in stomach contents analysis for prey identification, since otoliths are often the only identifiable components.

The Sparidae family (seabreams) is a ubiquitous taxon found in waters worldwide, especially in coastal ecosystems. Several important recreational and commercial fisheries are sustained by this teleost family. Sparidae are hosted by many marine habitats, from rocky to sandy substrates, at depths ranging from 0 to 500 m.

Among seabreams, species belonging to the *Pagellus* genus exhibit a wider geographical distribution. Blackspot seabream, *Pagellus bogaraveo* (Brünnich, 1768), axillary seabream, *Pagellus acarne* (Risso, 1826), and common pandora, *Pagellus erythrinus* (Linneus, 1758) are the most significant *Pagellus* species due to their high commercial value in the East Atlantic and the Mediterranean. Consequently, different fisheries target them.

These fishes demonstrate a cosmopolitan distribution in both hemispheres, with differences in relative abundance and frequency in Mediterranean areas, especially between western and eastern regions. Seabreams have different biological and ecological features. *P. acarne* and the *P. erythrinus* predominantly inhabit the continental shelf floor, while the continental slope is inhabited by *P. bogaraveo*^24^.

The *P. acarne* is commonly found in muddy and sandy substrates at depths between 40 and 500 m, with the highest frequency of occurrence between 40 and 100 m^25^. It is a carnivorous, euryphagous, and zooplanktivorous fish^26^. *P. acarne* exhibit protandric hermaphroditism; they are initially male with an immature ovarian zone, which subsequently becomes mature and functional as testicular regression occurs^25^. In most Atlantic fisheries (the northern Atlantic Algarve, Azores, and the Canary Islands), the *P. acarne* is a target species, especially among small-scale commercial fisheries^27^. In the Mediterranean, however, it is one of the principal by-catch species of artisanal vessels and trawlers. Little information exists about the status of stocks in Mediterranean regions, where the minimum landing size (17 cm) is the only management measure for the species.

*Pagellus erythrinus* is a demersal species with gregarious habits. It largely inhabits rocky and muddy-sandy substrates, exhibiting a high frequency of occurrence at depths between 20 and 300 m. *Pagellus erythrinus* is a generalist predator and a benthic feeder. It displays protogynous hermaphroditism^28,29^. *Pagellus erythrinus* has a high commercial value worldwide and is targeted by many commercial and artisanal fisheries, especially in the Atlantic and Mediterranean, where signs of overexploitation have been reported in many sub-regions^28^.

*Pagellus bogaraveo* is ubiquitous throughout the Mediterranean Sea, common in the western Mediterranean Sea, less common in the eastern Mediterranean Sea, and absent from the Black Sea^24^. It is also a species with high commercial value. *Pagellus bogaraveo* forms small schools above all substrata, near offshore banks, on seamounts^27,30^, and in cold-water reefs. *Pagellus bogaraveo* is a benthopelagic predator and a protandrous hermaphrodite (late first maturity as females). Juveniles live near the coast, whereas adults live on the continental slope at depths reaching 800 m. Adults reproduce all year round, with maximum reproduction varying according to region. This biological feature makes the species more sensitive to fisheries efforts^27,31^.

Although otoliths, particularly *sagittae,* are commonly used in several disciplines (e.g., systematics, auditory neuroscience, bioacoustics, fisheries biology, and ecology) to investigate fish biology and assess stocks, it is not yet fully understood how *sagitta* morphology varies inter- and intra-specifically regarding several ecological features of species.

Therefore, we investigate the intra- and inter-specific differences among *P. bogaraveo, P. acarne,* and *P. erythrinus* otoliths, to add to the knowledge base regarding the eco-morphology of *sagittae*.

To achieve an accurate description of the *sagittae* for each species, we first investigated existing differences in morphology and morphometry between juvenile and adult specimens and left and right *sagittae*. Moreover, it is of fundamental importance to answer still open questions, “What are the differences in *sagittae* between these congeneric species?” and “how these differences could be related to the eco-functional features and ecology of each seabream species by considering similarities and differences in lifestyle (e.g., feeding, bathymetric distribution, habitat, and locomotion)”.

Therefore, we examined a representative sample of *sagittae* from three congeneric seabreams, carefully analyzing the morphology, morphometry, and microstructure of the otoliths, and highlighting possible changes at different life stages and between left and right *sagittae*.

This study provides an accurate description of the *sagittae* of these seabream species, providing new data regarding the shape, using R software, and microstructure, using SEM imaging, of the *Pagellus* genus *sagittae*.

Moreover, deepening the knowledge about variations in *sagitta* eco-morphology and morphometry between and within these three congeneric species will lay the foundations for further studies concerning the functional-morphological aspects of fish otoliths. Concerning fisheries management, deeper knowledge about *sagittae* and their changes during fish growth can aid understanding of the stock structure, population connectivity, and dynamics of these species. This is essential for developing improved strategies for managing stocks with high commercial value.

Comparing the morphological features and morphometry of the *Pagellus* genus *sagittae* in the study area with data from other geographical areas could improve understanding of variations in *sagittae* morphology and morphometry in different geographical areas and habitats since these changes could be related to both genetic differentiation between populations and ecomorphological adaptation to different environments.

## Results

### Morphometric and shape analysis

The otoliths extracted from each specimen of the three studied species were examined and divided into juveniles and adults (when applicable). The 24 *P. bogaraveo* individuals were divided into seven juveniles (14 otoliths) and 17 adults (30 otoliths). The 37 *P. erythrinus* individuals were divided into eight juveniles (13 otoliths) and 29 adults (57 otoliths). All 24 *P. acarne* individuals belonged to adults (46 otoliths). The means and standard deviations of the measured morphometries are summarized in Table 1 (juveniles) and Table 2 (adults).

**Table 1:** Morphometric mean values with standard deviation (SD) and range of *P. bogaraveo* and *P. erythrinus* juvenile group individuals: OL (otolith length), OW (otolith width), OP (otolith perimeter), OS (otolith surface), SP (sulcus perimeter), SS (sulcus surface), SL (sulcus length), SW (sulcus width), CL (cauda length), CW (cauda width), OSL, (ostium length), OSW (ostial width), RW (rostrum width), RL (rostrum length), CI (circularity), RE (rectangularity), aspect ratio (OW/OL %), the ratio of otolith length to total fish length (OL/TL), percentage of the otolith surface occupied by the sulcus (SS/OS%), percentage of the sulcus length occupied by the cauda length (CL/SL%), percentage of the sulcus length occupied by the ostium length (OSL/SL%), rostrum aspect ratio (RW/RL%) and percentage of the rostrum length occupied by the otolith length (RL/OL%).

Otolith morphological characters (mm-mm^2^)	*P. bogaraveo* Mean ± SD	*P. bogaraveo* Min.–Max	*P. erythrinus* Mean ± SD (R otoliths)	*P. erythrinus* Min.–Max (Rotoliths)	*P. erythrinus* Mean ± SD (L otoliths)	*P. erythrinus* Min.–Max (L otoliths)
OL	5.75 ± 0.091	5.26–6.67	5.75 ± 0.198	5.44–5.96	5.41 ± 0.136	5.26–5.58
OW	3.77 ± 0.078	3.44–4.58	4.35 ± 0.394	3.85–4.88	3.93 ± 0.222	3.74–4.25
OP	17.97 ± 0.58	15.81–24.96	18.71 ± 2.409	15.98–22.44	17.53 ± 0.918	16.67–18.94
OS	15 ± 0.61	13.05–22.17	17.37 ± 2.070	14.54–19.8	15.38 ± 1.277	13.76–16.69
SP	11.66 ± 0.211	9.47–12.61	12.41 ± 0.189	12.14–12.64	12.14 ± 0.575	11.23–12.80
SS	2.46 ± 0.088	1.52–2.92	3.44 ± 0.036	3.40–3.49	3.49 ± 0.493	2.77–3.97
SL	4.60 ± 0.115	3.66–5.15	4.80 ± 0.217	4.62–5.16	4.82 ± 0.208	4.57–5.02
CL	2.44 ± 0.073	1.71–2.88	2.60 ± 0.228	2.29–2.92	2.44 ± 0.232	2.15–2.66
CW	0.90 ± 0.065	0.53–1.49	1.08 ± 0.255	0.85–1.42	0.97 ± 0.100	0.87–1.09
CP	5.81 ± 0.17	3.91–6.61	6.51 ± 0.300	6.13–6.97	6.08 ± 0.417	5.72–6.67
CS	1.15 ± 0.052	0.62–1.40	1.56 ± 0.117	1.35–1.64	1.47 ± 0.187	1.26–1.75
OSL	2.15 ± 0.093	1.68–2.67	2.20 ± 0.119	2.04–2.33	2.38 ± 0.034	2.34–2.43
OSW	1.19 ± 0.064	0.83–1.62	1.32 ± 0.128	1.22–1.54	1.28 ± 0.294	0.91–1.68
OSP	5.84 ± 0.121	5.23–6.72	5.90 ± 0.379	5.57–6.50	6.06 ± 0.350	5.57–6.50
OSS	1.31 ± 0.064	0.90–1.73	1.89 ± 0.141	1.79–2.13	2.02 ± 0.382	1.51–2.52
RW	2.21 ± 0.064	1.82–2.82	5.88 ± 0.486	5.56–6.72	2.32 ± 0.349	1.98–2.85
RL	1.36 ± 0.064	0.93–1.97	1.59 ± 0.286	1.35–2.07	1.02 ± 0.213	0.77–1.28
OP^2^/OS	21.57 ± 0.618	18.89–28.10	20.18 ± 3.057	17.57–25.44	20.14 ± 2.677	17.42–24.13
OS/(OLxOW)	0.68 ± 0.004	0.65–0.72	0.69 ± 0.007	0.68–0.7	0.71 ± 0.012	0.67–0.73
OW/OL %	65.73 ± 1.001	61.05–74.55	75.60 ± 4.46	70.81–81.92	73.53 ± 2.62	71.02–77.44
OL/TL	0.05 ± 0.000	0.05–0.06	0.058 ± 0.002	0.054–0.0038	0.054 ± 0.002	0.053–0.056
SS/OS %	1.6 ± 0.8	9.9–19	20.03 ± 2.47	17.29–23.49	22.59 ± 1.68	20.15–24.54
CL/SL %	53.3 ± 1.3	46.7–60.3	54.04 ± 2.96	49.56–56.64	50.56 ± 2.69	46.95–53.26
OSL/SL %	46.6 ± 1.38	39.6–53.2	45.96 ± 2.96	43.36–50.44	49.44 ± 2.69	46.72–53.05
RW/RL %	165 ± 5	131–194	233.58 ± 24.58	197.40–256.63	229.78 ± 23.61	198.01–257.33
RL/OL %	23.4 ± 1.15	15.8–34.9	17.73 ± 1.99	14.94–19.28	18.93 ± 3.94	14.04–22.97

The morphometric data of *Pagellus bogaraveo* shown in the table relate only to the left otolith since no significant difference was found between the left (L) and right (R) sides.

**Table 2:** Morphometric mean values with standard deviation (SD) and range of *P. acarne*, *P. bogaraveo,* and *P. erythrinus* adult group individuals: OL (otolith length), OW (otolith width), OP (otolith perimeter), OS (otolith surface), SP (sulcus perimeter), SS (sulcus surface), SL (sulcus length), SW (sulcus width), CL (cauda length), CW (cauda width), OSL (ostium length), OSW (ostium width), RW (rostrum width), RL (rostrum length), CI (circularity), RE (rectangularity), aspect ratio (OW/OL%), the ratio of otolith length to total fish length (OL/TL), percentage of otolith surface occupied by the sulcus (SS/OS%), percentage of the sulcus length occupied by the cauda length (CL/SL%), percentage of the sulcus length occupied by the ostium length (OSL/SL%), rostrum aspect ratio (RW/RL%) and percentage of the rostrum length occupied by the otolith length (RL/OL%). (R = right, L = left).

Otolith morphological characters (mm-mm^2^)	*P. bogaraveo* Mean ± SD	*P. bogaraveo* Min.–Max	*P. erythrinus* Mean ± SD (R otoliths)	*P. erythrinus* Min.–Max (R otoliths)	*P. erythrinus* Mean ± SD (L otoliths)	*P. erythrinus* Min.–Max (L otoliths)	*P. acarne* Mean ± SD (R otoliths)	*P. acarne* Min.–Max (R otoliths)	*P. acarne* Mean ± SD (L otoliths)	*P. acarne* Min.–Max (L otoliths)
OL	9.81 ± 0.34	7.40–13.90	10.04 ± 1.092	7.37–11.95	9.83 ± 1.175	7.46–12.16	9.67 ± 0.875	8.11–11.25	9.81 ± 1.332	4.98–11.24
OW	6.06 ± 0.19	4.57–8.29	7.76 ± 0.865	5.89–9.14	7.21 ± 0. 894	5.44–8.76	4.99 ± 0.500	4.09–5.70	5.21 ± 0.901	4.38–9.10
OP	29.37 ± 1.06	21.64–43.63	31.10 ± 3.628	24.56–11.95	31.19 ± 3.273	26–37.94	28.77 ± 3.69	21.85–38.22	30.12 ± 3.390	23.10–35.84
OS	41.4 ± 2.85	24.23–76.71	52.59 ± 10.56	31.25–72.21	49.40 ± 10.943	27.50–69.45	32.79 ± 5.381	22.47–41.49	34.66 ± 4.682	26.37–41.54
SP	19.83 ± 0.51	14.01–25.24	21.61 ± 3.507	12.26–26.47	21.91 ± 2.750	16.30–27.27	20.06 ± 2.605	15,01–25,12	20.52 ± 2.535	16.49–25.21
SS	6.12 ± 0.27	3.61–9.49	11.72 ± 3.201	5.58–18.42	10.56 ± 2.750	5.77–15.81	7.38 ± 1.562	4.95–9.98	7.87 ± 1.608	5.55–10.41
SL	8.01 ± 0.20	5.95–9.69	8.43 ± 1.129	5.95–11.18	8.71 ± 1.101	6.33–10.85	7.49 ± 1.739	3.05–10.22	7.98 ± 1.874	3.13–10.47
CL	4.189 ± 0.131	3.03–5.59	4.51 ± 0.580	3.26–5.47	4.70 ± 0.613	3.52–5.74	4.03 ± 1.042	1.38–5.61	4.55 ± 1.203	1.26–5.96
CW	1.50 ± 0.063	0.77–2.13	2.370 ± 0.584	1.41–3.94	1.73 ± 0.255	1.25–2.40	2.19 ± 0.997	1.30–5.44	1.91 ± 0.940	1.25–5.00
CP	9.93 ± 0.29	7.32–12.74	11.20 ± 2.730	2.34–14.97	11.66 ± 1.454	9.03–14.03	10.69 ± 1.565	8.12–13.47	11.33 ± 1.369	13.37–8.60
CS	2.95 ± 0.14	1.46–4.35	5.41 ± 1.524	2.85–8.28	4.74 ± 1.181	2.78–7.25	4.07 ± 1.080	2.15–5.76	4.39 ± 0.890	3.08–5.97
OSL	3.82 ± 0.10	2.61–4.92	3.913–0.685	2.69–6.29	4.01 ± 0.610	2.81–5.26	3.46 ± 0.795	1.48–4.68	3.43 ± 0.756	1.87–4.80
OSW	1.70 ± 0.080	1.03–3.26	3.053 ± 2.191	1.30–12.86	2.10 ± 0.391	1.45–2.93	2.06 ± 0.611	1.43–3.89	2.19 ± 0.460	3.40–1.40
OSP	9.89 ± 0.29	6.46–14.24	10.41 ± 1.401	7.20–12.71	10.26 ± 1.545	7.27–13.23	9.37 ± 1.437	6.88–12.25	9.19 ± 1.362	6.71–11,93
OSS	3.16 ± 0.15	1.88–5.65	6.31 ± 1.800	2.51–10.14	5.82 ± 1.708	2.93–9.55	3.31 ± 0.793	2.14–4.76	3.48 ± 0.837	5.20–1.96
RW	3.26 ± 0.08	2.31–4.42	9.83 ± 1.805	6.20–13.30	3.67 ± 0.537	2.87–5.04	3.17 ± 0.520	2.25–4.05	3.42 ± 0.510	2.57–4.34
RL	2.29 ± 0.080	1.61–3.18	4.37 ± 1.748	1.41–8.23	1.73 ± 0.380	1.13–2.77	2.58 ± 0.400	1.77–3.22	2.97 ± 0.049	2.29–4.33
OP^2^/OS	21.39 ± 0.26	19.07–25.16	18.57 ± 1.271	16.61–22.65	20.21 ± 3.27	17.54–33.28	25.46 ± 3.917	19.94–36.90	26.35 ± 3.892	20.24–34.67
OS/(Ol × OW)	0.67 ± 0.006	0.55–0.73	0.67 ± 0.023	0.62–0.72	0.69 ± 0.66	0.42–0.80	0.67 ± 0.025	0.64–0.73	0.69 ± 0.021	0.63–0.72
OW/OL%	62 ± 0.5	57–69	77.45 ± 5.06	69.38–91.22	73.41 ± 2.53	68.53 -80.04	51.71 ± 3.49	47.01–59.43	56.02 ±27.21	44.27–182.66
OL/TL	0.04 ± 0.001	0.04–0.06	0.054 ± 0.0038	0.048–0.062	0.053 ± 0.005	0.04–0.06	0.047 ± 0.005	0.035–0.054	0.047 ± 0.006	0.024–0.055
SS/OS%	15 ± 0.5	9.4–18.9	22.15 ± 2.34	16.10–25.52	21.44 ± 3.86	16.03–38.36	22.50 ± 2.80	16.39–27.08	22.59 ± 2.60	16.57–27.78
CL/SL%	52 ± 0.6	47–63	53.69 ± 3.22	43.76–58.86	54.05 ± 2.98	44.30–60.72	53.44 ± 4.38	41.09–58.09	56.28 ± 5.82	39.22–63.82
OSL/SL%	47 ± 0.6	36–52	46.31 ± 3.22	56.24–41.14	45.95 ± 2.98	39.28–55.70	46.56 ± 4.38	41.91–58.91	43.73 ± 5.82	36.18–60.78
RW/RL%	144 ± 2.8	120–186	230.51 ± 38.28	91.43–317.81	215.95 ± 23.33	161.49–254.20	125.15 ± 22.76	79.47–152.30	115.85 ± 12.44	87.70–132.92
RL/OL%	23.7 ± 0.6	18–30	17.42 ± 3.80	9.31–30.07	17.60 ± 3.02	10.97–24.80	26.78 ± 4.23	20.39–36.57	30.87 ± 6.78	22.07–57.14

The *P. bogaraveo* specimens exhibited an elliptical otolith shape with crenate margins, developmentally increasing margin regularity, notch depth, and antirostrum length. The t-test performed on otolith morphometrical parameters did not reveal differences between the right and left *sagittae* in the two size groups analyzed (Supplementary Table S1).

The differences between adults and juveniles were observed in the shape and size of the rostrum, the shape and borders of the ventral and dorsal margins, and the proportions of otolith length to fish length and otolith width to otolith length. In the otoliths of juveniles, dorsal and ventral margins were lobed, and the rostrum was shorter, broader, and rounder. The *sulcus acusticus* occupied a greater sagittal area compared to adult otoliths, with the *cauda* larger than the ostium. In adult specimens, the sulcus penetrated deeper into the sagitta’s carbonate structure compared to juveniles. Significant differences were detected in *sagitta* aspect ratio (OW/OL %), *sagitta* length to total fish length ratio (OL/TL), and rostrum aspect ratio (RW/RL %) between juveniles and adults (Supplementary Table S1).

As in *P. bogaraveo,* in *P. acarne* adult specimens, the *sulcus acusticus* penetrated deeper into the *sagitta* structure (Figs. 2d–e; 4e–f). In the *P. acarne* specimens, significant differences in rostrum aspect ratio (RW/RL %) were detected between right and left *sagittae* (Supplementary Table S1). A significant negative correlation was found between *sagitta* aspect ratio (OW/OL %) and fish total length (TL), while a positive correlation was observed between relative sulcus area percentage (SS/OS %) and fish weight (Supplementary Table S2).

The *P. erythrinus* specimens displayed a pentagonal otolith shape, highlighted by high rectangularity, with a high circularity value. In juvenile specimens, the rostrum and sagittal width increased in relation to fish length and weight, while the *sagitta* length values did not varied significantly between juveniles and adults, highlighting an exponential increase in fish length compared to sagittal length. In adult *P. erythrinus* specimens, the sulcus did not penetrate as deeply as in the other two seabreams species (Fig. 3d–f). *Pagellus erythrinus* individuals did not display significant differences between juveniles and adults, although significant differences in circularity (OP^2^/OS), rectangularity (OS/(OL × OW)), and *sagitta* aspect ratio (OW/OL %) were detected between right and left *sagittae* (Supplementary Table S1).

In the juvenile group, a negative correlation between *sagitta* length to total fish length ratio (OL/TL), fish weight, and total length was highlighted. A statistically significant positive correlation was observed between rostrum aspect ratio (RW/RL %) and fish weight, *sagitta* aspect ratio (OW/OL%) and fish weight, and *sagitta* aspect ratio (OW/OL%) and fish total length (Supplementary Table S2).

The negative correlation between *sagitta* length to total fish length ratio (OL/TL), fish weight, and total length was also observed in the adult group, whereas a significant positive correlation was detected for relative sulcus area percentage (SS/OS %) and fish weight, and relative sulcus area percentage (SS/OS%) and fish total length (Supplementary Table S2).

The t-test performed on juvenile specimens of *P. bogaraveo* and *P. erythrinus* did not show significant differences only for percentage of the sulcus length occupied by the cauda length (CL/SL%), percentage of the sulcus length occupied by the ostium length (OSL/SL%), and percentage of *sagitta* length to total fish length ratio (OL/TL %). A one-way ANOVA performed on the morphometrical parameters of adult samples, showed the following significant differences: circularity (OP^2^/OS), *sagitta* length to total fish length ratio (OL/TL), *sagitta* aspect ratio (OW/OL%), relative sulcus area percentage (SS/OS%), percentage of the otolith length occupied by rostrum length (RL/OL%), and rostrum aspect ratio (RW/RL%) between *P. bogaraveo* and *P. erythrinus*; circularity (OP^2^/OS), *sagitta* length to total fish length ratio (OL/TL), *sagitta* aspect ratio (OW/OL%), relative sulcus area percentage (SS/OS%), percentage of the otolith length occupied by rostrum length (RL/OL%), rostrum aspect ratio (RW/RL%), percentage of the sulcus length occupied by the cauda length (CL/SL%) and percentage of the sulcus length occupied by the ostium length (OSL/SL%) between *P. bogaraveo* and *P. acarne*; and circularity (OP^2^/OS), *sagitta* length to total fish length ratio (OL/TL), *sagitta* aspect ratio (OW/OL%), percentage of the otolith length occupied by rostrum length (RL/OL %) and rostrum aspect ratio (RW/RL %) between *P. erythrinus* and *P. acarne* (Supplementary Table S1).

Interestingly, the first two axes (PC1 and PC2) of the PCA plot showed slight separation in the *sulcus acusticus* parameters between the fish species analyzed. In particular, PC1 (74%) separated *P. erythrinus* sulcus acusticus parameters from *P. bogaraveo* and *P. acarne* parameters, which overlapped on the left side of the diagram along PC2 (26%). As shown in the LDA plot, *Pagellus* spp. resulted well separated (Supplementary Figure S1a, b).

**Figure 1:**
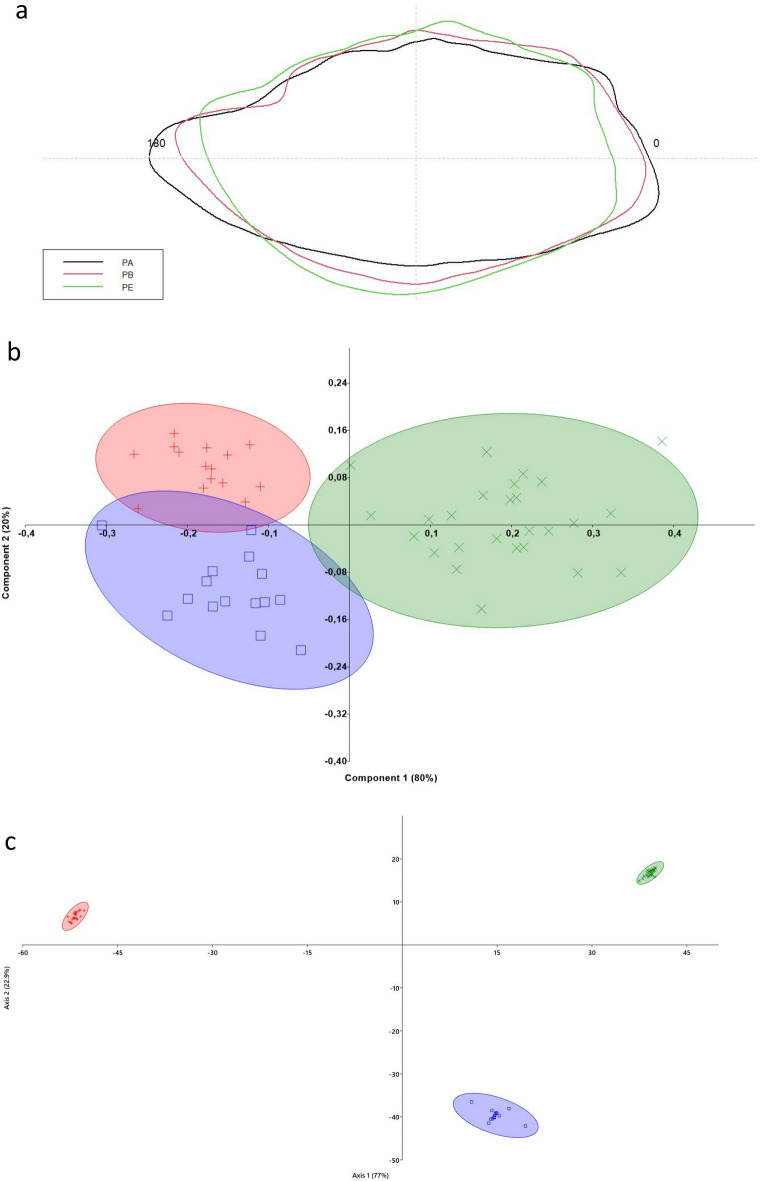
(**a**) Mean shapes of otolith contours. PA is *Pagellus acarne,* PB is *Pagellus bogaraveo*, and PE is *Pagellus erythrinus.* (**b**) Principal component analysis plot (PC1 versus PC2) of the otolith contours computed between the species analyzed. The PCA was based on wavelet Fourier descriptors, with 95% probability ellipses shown. (**c**) Linear Discriminant Analysis between species, calculated on elliptic Fourier descriptors. Ellipses include 95% confidence interval.

The mean shape of otoliths differed significantly between *P. bogaraveo*, *P. erythrinus*, and *P. acarne* (*P* < 0.001), although minor differences were observed between *P. bogaraveo* and *P. acarne.* The otolith contours are shown in Fig. 1a. The first two axes (PC1 and PC2) of the PCA plot showed a separation of otoliths contours between the three fish species. In particular, PC1 (80%) separated *P. erythrinus* otolith shape from that of *P. bogaraveo* and *P. acarne*, which overlapped on the left side of the diagram along PC2 (20%) (Fig. 1b). Marked differences in the otoliths shape have also been confirmed by LDA. From the LDA plot of the first two discriminant functions, we can see that *Pagellus* species were quite well separated (Fig. 1c).

### Scanning electron microscopy (SEM) analysis

Among the otoliths in all the examined species, SEM showed clear changes in the shape, size, and direction of the external textural organization of the *sulcus acusticus* and differences in the surface of the *crista* superior and inferior between juvenile and adult individuals at the intra-specific level.

**Figure 2:**
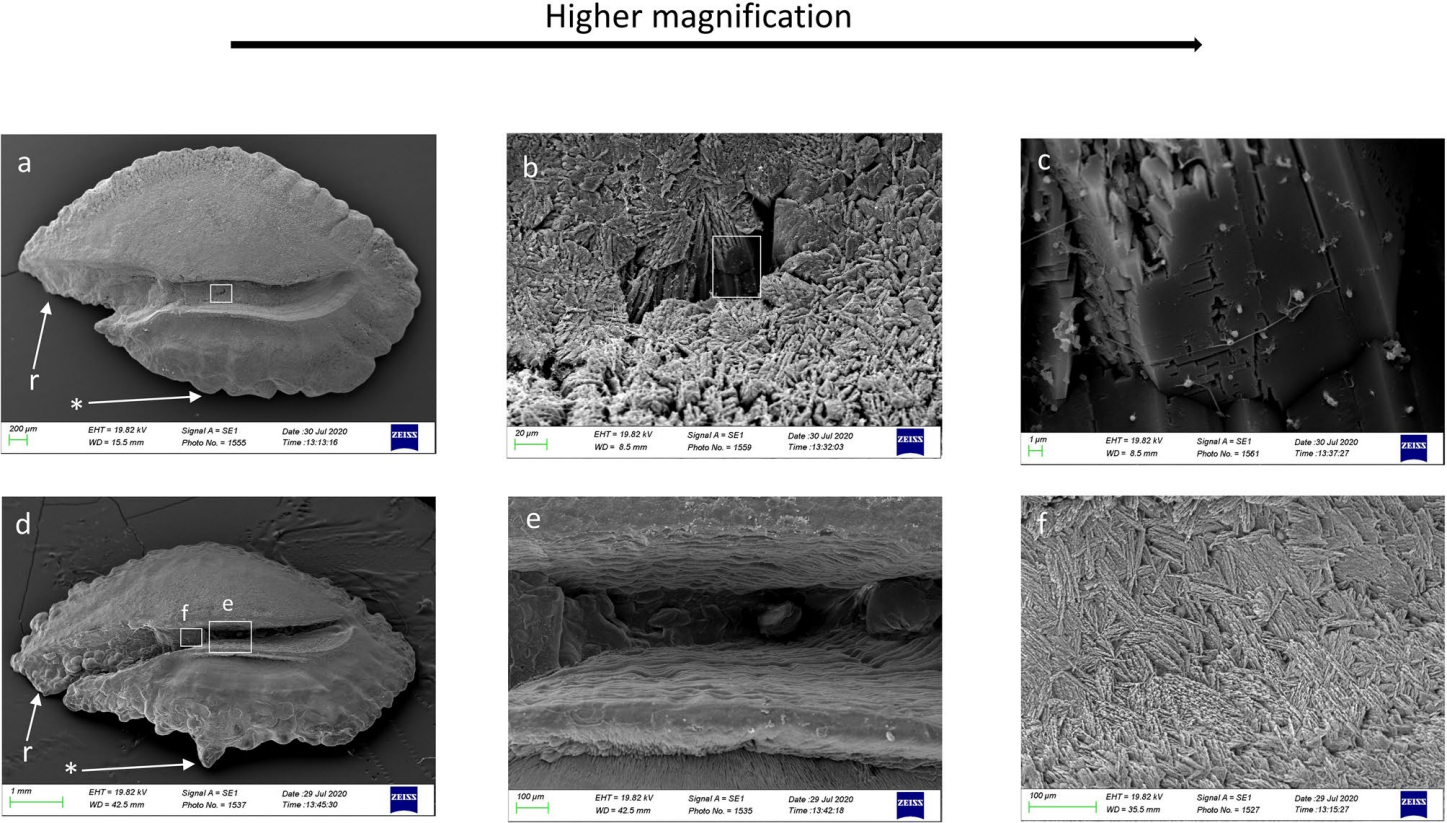
SEM images of the proximal surface of *P. bogaraveo*: juvenile (**a**) and adult (**d**) right *sagitta*, with details of a tip on sulcus acusticus surface (**b**–**c**), and details of external textural organization and crystalline structure (**f**) of caudal surface (**e**). (r) Indicates the rostrum and (*) indicates the dorsal rim.

**Figure 3:**
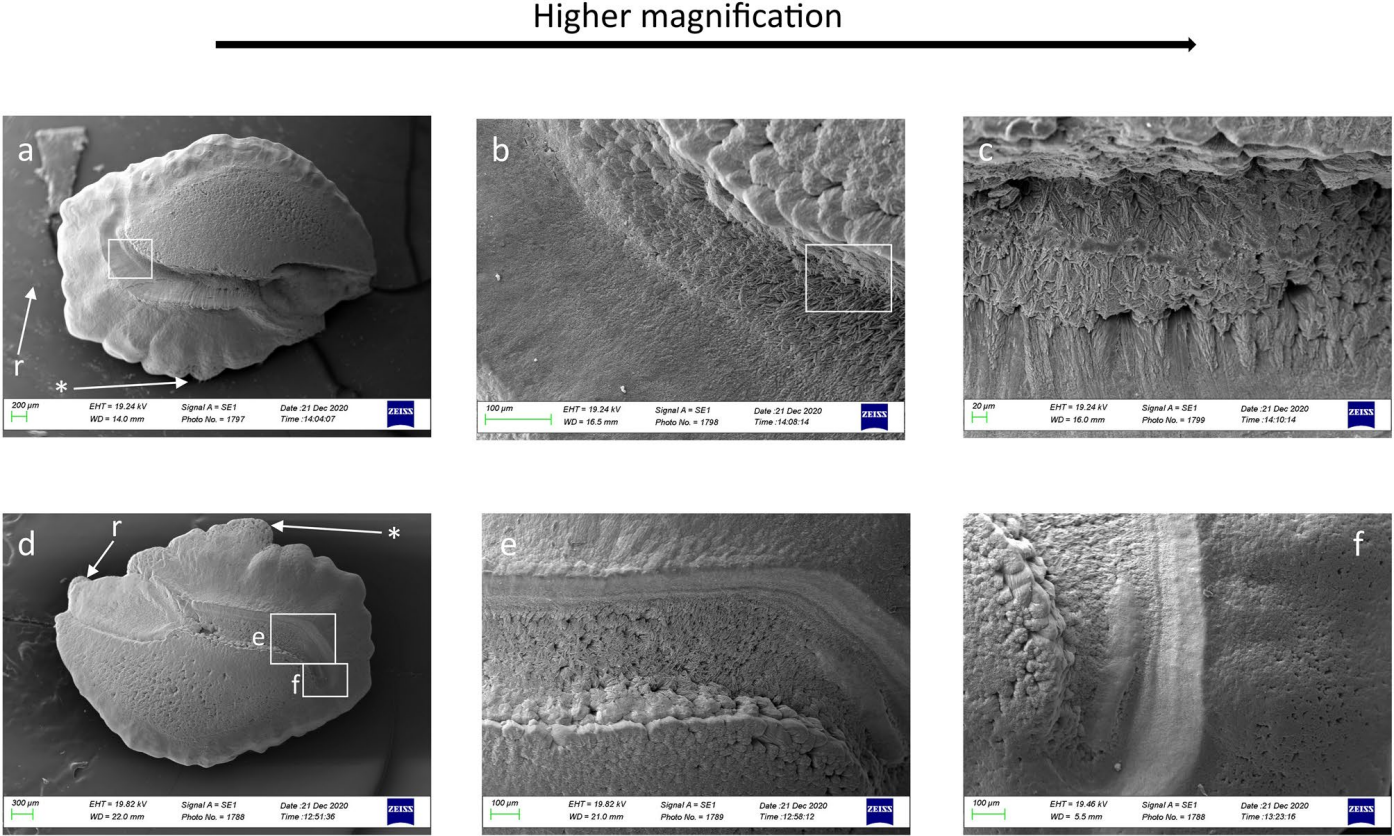
SEM images of the proximal surface of *P. erythrinus*: adults (**d**) and juveniles (**a**) otoliths left sagittae, with details of sulcus acusticus (**b**, **c**, **e**, **f**). (r) indicates the rostrum and (*) indicates the dorsal rim.

In juveniles, the *crista* superior and inferior sloped gently toward the *sulcus acusticus* depression, with an almost flat surface (Figs. 2a, 3a). The *sulcus* surface appeared smoother than in adults, with several tips distributed over the entire sulcal surface (Figs. 2b, c, 3c). In juveniles, the external textural organization of the sulcus was composed of smaller and thin crystals sometimes melted together or embedded in organic materials. In comparison, however, the crystals in adult specimens had become larger and thicker (Figs. 2b, f, 5a–h). The crystals of juveniles were grouped, with rounded edges, and slightly orientated in the vertical and oblique planes, with the long axis of crystals following the incremental growth direction of the otoliths (from the nucleus to the outer edge of the growth) (Fig. 5a, b, e, f). Not all the crystals had the same shape and external 3D organization as in the adults; they had a smoother surface with a more compact structure.

**Figure 4:**
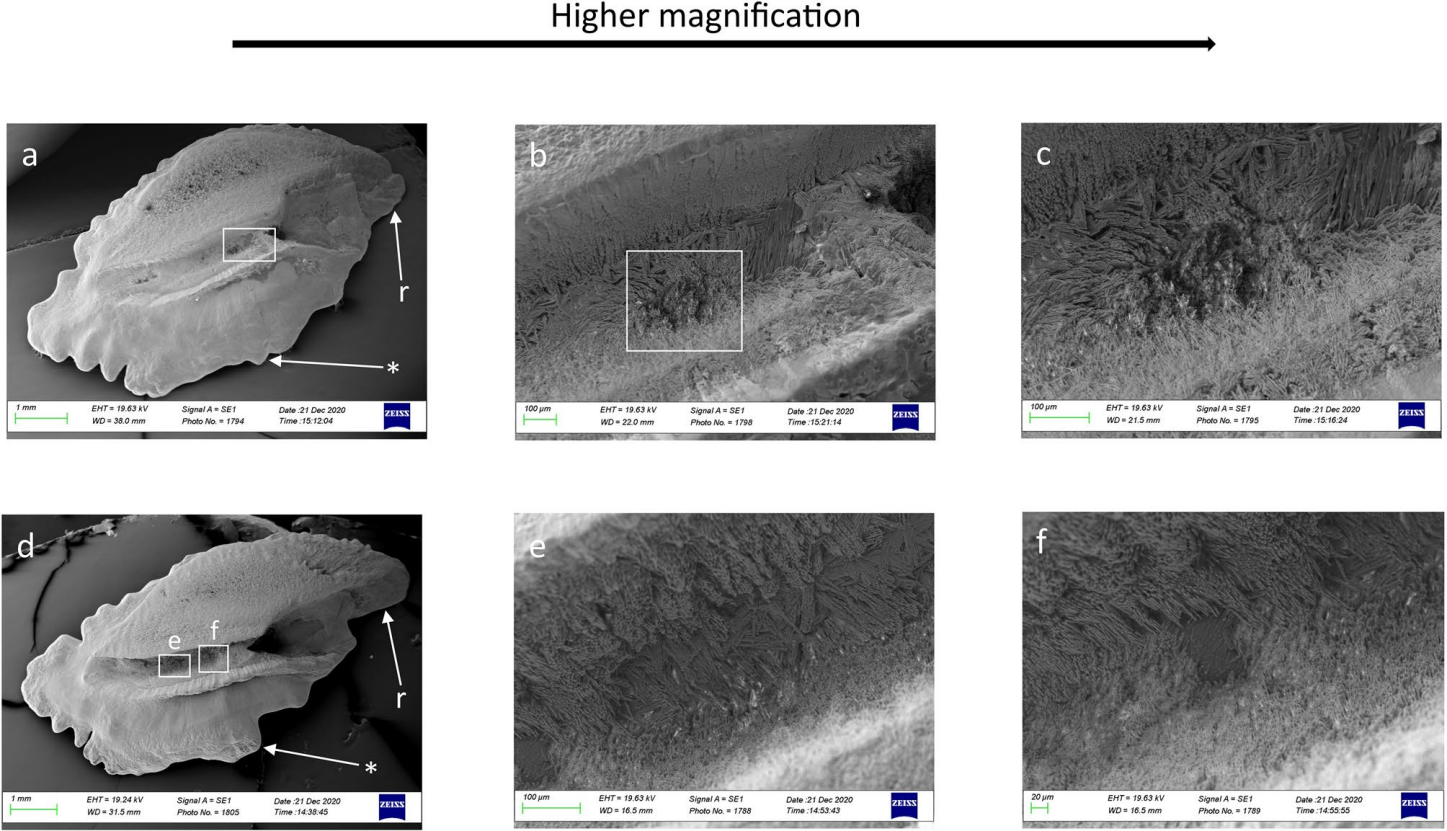
SEM images of the proximal surface of *P. acarne*: adult otoliths left sagitta (**a**–**d**), with details of sulcus acusticus (**b**–**e**), and external textural organization (**c**–**f**). (r) indicates the rostrum and (*) indicates the dorsal rim.

**Figure 5:**
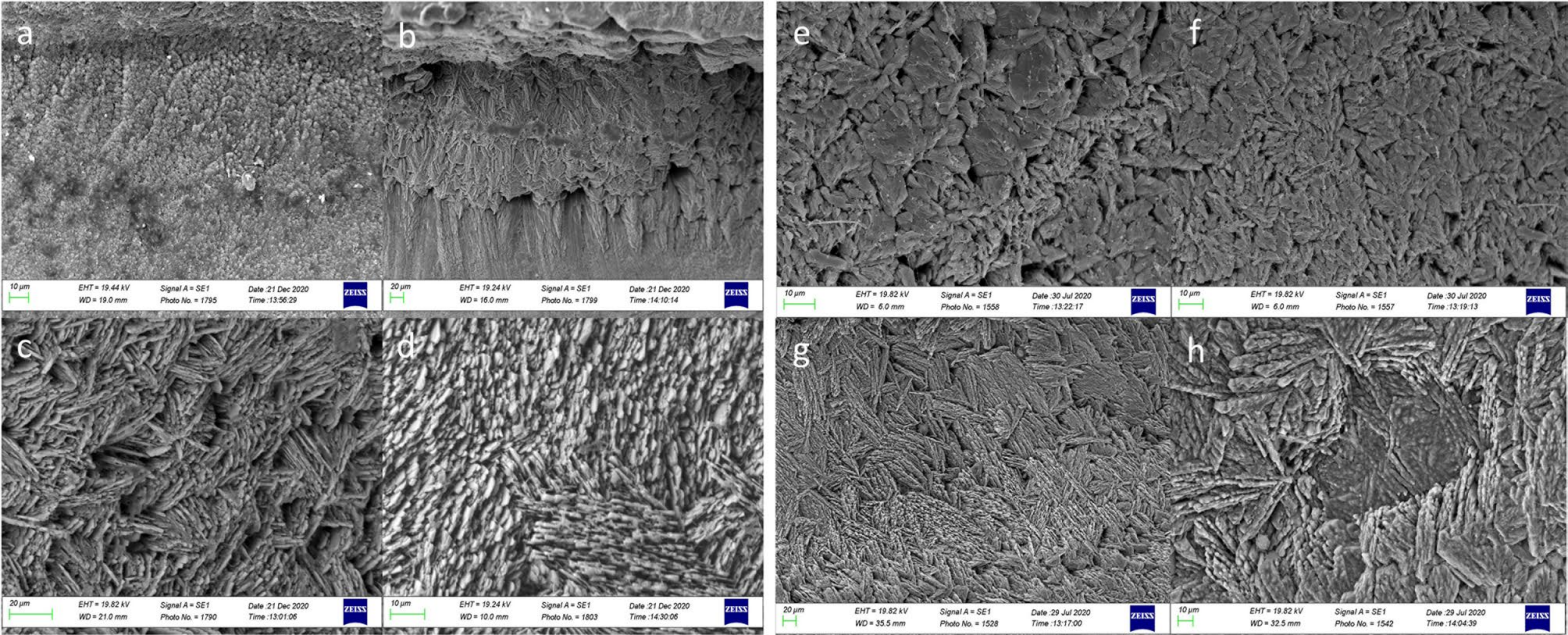
SEM images of the crystalline structure of *P. bogaraveo*, juveniles (**a**, **b**) and adults (**c**, **d**), and *P. erythrinus*, juveniles (**e**, **f**) and adults (**g**, **h**) *sagittae.*

Regarding adult specimens, the *crista* surface steeply declined towards the *sulcus*, with a hollow preceding the sulcal depression (Figs. 2d, 3d, 4a–d). The external sulcal structure was more complex with a more textured, rougher surface than in juveniles. The crystals were narrowed, with a prismatic shape. They had sharp edges, were almost equally sized, longer, and had a more chaotic orientation than in juvenile individuals (Fig. 5c, d, g, h).

Our results showed that the sulcus of *Pagellus* individuals from the south Tyrrhenian Sea was heterosulcoid. Furthermore, a greater size difference in the ostium and cauda was observed in adult *P. erythrinus* and *P. bogaraveo* compared to juveniles. Generally, the cauda was larger than the ostium and markedly different in shape. Despite the ostium, cauda growth during fish development indicated a most pronounced heterosulcoid character in adult individuals than in juveniles.

The macroscopic structure and shape of *sulcus acusticus* displayed in the SEM images exhibited inter-specific differences between the three congeneric seabreams. The *ostium* of *P. acarne* otoliths was deeper than that in the other congeneric species, and it displayed, with those of *P. bogaraveo,* according to with the *ostium* and *cauda* shape classification^1^, a funnel-like shape with concave *ostium* walls that expand and broaden anteriorly from the region of confluence with the *cauda.* Unlike the other seabreams examined in this study, the shape of the *ostium* in the *P. erythrinus sulcus acusticus* was more rectangular, with a markedly tubular shape, and the cauda was distinctly curved, especially in juvenile specimens.

## Discussion

### Intra- and interspecific differences: comparison with former studies on *Pagellus* species and other fish species

To understand the relationship between function, shape, and the environment, it is essential to include the morphological variability of otoliths, considering biological and environmental variability leads to otolith shape heterogeneity through morpho-functional adaptation to different habitats. Several authors have highlighted changes in otolith shape between species and, in many cases, among populations of the same species (e.g., herrings, salmonids, and lutjanids). The intra-specific variability of otolith morphology and shape are the basis of stock separation and assessment and is related, especially in *sagittae*, with environmental (e.g., water temperature, salinity, and depth) and biological factors (e.g., sex, ontogeny, and genetic variability)^20^.

The analysis of the three *Pagellus* species revealed that otolith morphology and morphometry did not follow those described in a previous study^1^ conducted in the western Mediterranean Sea and the Atlantic Ocean in term of rectangularity, circularity, *sagitta* aspect ratio and *sagitta* length to total fish length ratio. Although the images provided in our study closely resembled those from research in other geographical areas, the morphometric measures (obtained according to the procedures and methods described in the previous literature^1,20,23,32^) exhibited several differences. Considering the scale of our study compared to previous studies, it is difficult to provide an entirely valid comparison; the differences in *sagitta* morphology and morphometry could have been triggered by biotic and abiotic parameters (e.g., temperature, salinity, genotype, habitat type, differences in food quality and quantity)^13,33–35^. Such environmental and genetic factors may be primary drivers of otolith morphometry and morphology among fishes in different habitats. Therefore, detected shape differences are at the basis of fish stock differentiation^36^.

Our results indicate that the min–max circularity and rectangularity of *P. bogaraveo* from the Southern Tyrrhenian Sea differ from those calculated in a previous study^1^ in the western Mediterranean Sea, and the north and central-eastern Atlantic ocean. Moreover, the increase in circularity in larger specimens, confirms a greater tendency toward circular than elliptical otolith shape in southern Tyrrhenian Sea species compared to those in other Mediterranean and Atlantic areas.

Despite statistical differences and correlations in this study supported the hypothesis that some changes in *sagitta* morphology are related to fish size differences, several aspects and studies should be performed to better understand this relation. The negative correlation between the ratio of *sulcus acusticus* surface to the entire *sagitta*, rostral morphology, and the increase in specimen’s size was related to the expansion in the length and surface of the entire sagitta and rostral area in larger specimens. These features, with no statistical relevant increment in *sulcus acusticus* surface and increased *rostrum* length, could be correlated with more pronounced peripheral *sagitta* growth in this species. *Sagitta*, in fact, after fish pelagic phase, might increases its surface in the rostral area and the margins. Since the present study did not take into account ontogenetic stages and specimens age, it is hard to relate this result with *sagitta* and *sulcus acusticus* growth. But reading this increase by an ecological point of view, it could be related to the lifecycle of the species. During the juvenile stage, in the early stage of pelagic life, the species inhabits shallow water. Adults inhabit deep-water environments, migrating down the continental slope to a depth of 800 m after the juvenile stage. These changes in habitat might be the cause of morphological variations in the *sagittae,* highlighting the relationship between *sagitta* features and environmental and biological factors.

The *P. acarne* specimens demonstrated the highest number of morphometrical parameters that did not follow those of the same species described in a previous study (i.e., circularity, rectangularity, sagitta length to total fish length ratio, and *sagitta* aspect ratio)^1^. These morphometrical changes are reflected in otolith shape. The otoliths from specimens in our study were largely circular, with highly irregular margins and a *rostrum* that varied in length and width through the left and right *sagitta*, as indicated by the significant differences in rostrum aspect ratio values.

The morphometrical results in *P. erythrinus* revealed differences in circularity, rectangularity, *sagitta* length to total fish length ratio and *sagitta* aspect ratio compared to a previous study^1^ in the western Mediterranean Sea and north-central eastern Atlantic ocean.

The *P. erythrinus* specimens were characterized by a pentagonal otoliths shape, and increased circularity compared to the same species from other areas. The results also indicated small differences between the left and right *sagitta*. This small differences were previously described in other Mediterranean sub-areas, for example, otolith width values in *P. erythrinus* specimens collected in the Gulf of Tunisia^37,38^.

As said above for *P. bogaraveo*, it is hard to relate the differences between juveniles and adults with fish growth due to the absence in present paper of ontogenetic and age analysis. The higher width than length, demonstrated by min–max width values in Tables 1 and 2, in *sagittae* of adults *P. erythrinus* specimens could be correlated with an exponential increment in fish size compared to the sagittal length. Further analyses on ontogenetic development of this species are required to better define the *sagitta* growth related to fish growth.

The increase in *sulcus acusticus* surface exhibited in the adult specimens could be correlated with feeding habits; during its adult life, this species is a benthic feeder and inhabits deeper environments than juveniles^28,29^.

Although meaningful lateral dimorphism of the *sagittae* was detected only in flatfish, statistical analysis revealed several small differences between the left and right *sagitta* in *P. erythrinus* and *P. acarne*, as previously described in other round fish species, such as *Chelon ramada* (Risso, 1827)^40^, *Diplodus annularis* (Linnaeus, 1758)^41^, *Diplodus puntazzo* (Walbaum, 1792)^42^, *Clupea harengus (*Linnaeus, 1758)^43^, and *Scomberomorus niphonius* (Cuvier, 1832)^44^.

Our study confirmed slight differences between width values in left and right *sagitta* previously described in *P. erythrinus* and extend the differences to other parameters, such as circularity and rectangularity (Tables 1, 2). Concerning *P. acarne,* however, marginal differences between the left and right *sagittae* were observed for the first time.

This slight differences are supported by the literature concerning genetic and environmental stressors^41^. Since the functional morphology of otoliths is not completely understood, it is difficult to find a direct link between these small differences and the ecology of the species. However, several eco-functional factors, such as feeding behavior, deserve attention as fundamental for a better understanding of the relationship between otolith features and species habitat. For example, *P. erythrinus* largely preys on strictly benthic organisms, such as polychaetes, brachyuran crabs, and benthic crustaceans. Most of these species frequently escape predators by hiding under the sandy substrate. Other Sparidae (*Lythognathus mormyrus,* Linnaeus, 1758) feed on benthic fauna, engulfing sediment and filtering it in the buccal cavity, demonstrated by the high percentage of detritus and benthic remains (e.g., scales, urchin spines, and benthic foraminifers) in the gut and stomach contents^29^. To engulf sediment, *P. erythrinus* performs a particular movement with the head and body, laterally shifting and pushing forward, to dig the bottom sand and reach prey. This kind of behavior, common in all benthopelagic species with the same feeding habits, could influence the *sagitta* growth and morphology, triggering small differences between the left and right *sagitta*. Further studies on this and other species with this behavior (e.g., *L. mormyrus*) are necessary to confirm this hypothesis.

Concerning inter-specific differences in *sagitta* morphology among the three species, it is difficult to read the results obtained in this study eco-morphologically since an insufficient understanding of the functional morphology and physiology of otoliths prohibits a direct relationship, valid for all the species, between eco-functional features and otolith morphology. Nevertheless, as expected, the shape analysis (Fig. 1) revealed clear differences between the three congeneric species. Considering several ecological, functional, and biological features in each species, the results have demonstrated a *sagitta* morphology that could be in accordance with the ecology and lifestyle of these three congeneric seabreams.

### Relationship between otolith morphology and ecology/lifestyle

The *sagittae* of *P. acarne* exhibited a shape resembling those in other pelagic species, with a long *rostrum* and the entire *sagitta* elongated and narrower than those in other two seabream species. The species that show the most pelagic habits, with largely planktivorous feeding at a small size, adapt also to benthopelagic feeding activity in adult life. The statistically relevant similarity found in *P. bogaraveo* could be proof of the ecomorphological adaptation of *sagittae* to pelagic and demersal environments. This hypothesis may be confirmed by marked differences in shape compared to those in *P. erythrinus,* which is the most benthic among the three species.

*Pagellus erythrinus* was the species with the shortest *rostrum.* It also has the most benthic habits, largely preying on epibenthic and infaunal species. Moreover, its ecology and life cycle differ among the three species under study since they are strictly related to the benthic environment. This lifestyle could be in accordance with the differences observed in the shape analysis results. The *sagitta* contours appeared more circular and wider than those in the other two species. The PCA and LDA also confirmed the most difference in shape among the three species.

The species with the most marked antirostrum and *sagitta* shape was *P. bogaraveo*, which is a cross between the other two congeneric species. *Pagellus bogaraveo* is a demersal species, which inhabits the deep biocenosis and feeds in both benthic and mesopelagic environments. Furthermore, the ecology of this species could support the *sagitta* shape described in our study^27,30^.

Otolith morphology and morphometry in congeneric *Pagellus* species described in this study has followed the relationship between sagittal parameters, habitat, and depth described in previous literature^15^. According to several authors, the percentage of species with large otoliths increases with depth, except for abyssal depth. The specimens of *P. bogaraveo* analyzed in this paper (especially adult individuals) had larger otoliths than the other two *Pagellus* species due to their demersal habits (they inhabit the continental slope to a depth of 800 m). A larger sagitta is essential in demersal environments to compensate for light reduction by providing improved acoustic communication, sound perception^15,45^, and a sense of equilibrium^46^.

### Sulcus shape

Considering the *sulcus acusticus,* in the otolith atlas for the western Mediterranean Sea and Atlantic ocean^1^, studies describing and comparing otoliths^10^ and the diversity and variability of otoliths in teleost fishes^9^, the sulcus in *P. bogaraveo, P. acarne* and *P. erythrinus* was described as heterosulcoid, with an ostium shorter than the cauda and a long, narrowed rostrum, especially in adult *P. bogaraveo* and *P. acarne* individuals. Heterosulcoid otoliths were also observed in south Tyrrhenian Sea *Pagellus* individuals, with marked differences between juvenile and adult specimens. In a demersal species, such as *P. bogaraveo,* juveniles live in shallow, coastal water. Once adults, they inhabit deeper water (to a depth of 800 m). Changes in the crystalline and morphological structure of *sulcus acusticus* between juveniles and adults reflect this species’ need to adapt to deeper environments with less light.

The results indicate that in *P. bogaraveo,* the *sulcus acusticus* does not differ in surface between juvenile and adult specimens. This feature could be correlated with earlier *sulcus acusticus* development in this species*,* compared to *P. erythrinus* and *P. acarne,* emphasizing the role of the *sulcus acusticus* in this demersal species^48,49^. This might also confirm the strict correlation between biological and environmental factors and *sagitta* morphology in studied seabreams species.

Another morphological feature of the *sulcus acusticus,* which might support the ecology of the species, is the deep ostium and cauda. In adult specimens of *P. bogaraveo* and *P. acarne,* the *sulcus* structure deeply penetrated in the *sagitta* carbonate structure. Conversely, in adult *P. erythrinus* specimens, the sulcus did not penetrate as deeply as in the other two seabream species. This sulcal feature could correspond with the ecology and feeding behavior of *P. erythrinus*, which specializes in benthic strategies, including small differences between left and right *sagitta* and the absence of the notch and antirostrum in *sagittae*.

Although the deeper *sulcus acusticus* in *P. bogaraveo* and *P. acarne* might be linked to depth distribution, as in *P. bogaraveo,* it may also correspond with high mobility related to feeding behavior, as in both *P. bogaraveo* and *P. acarne.* The different depths of *sulcus acusticus* can change the thickness of the otolithic membrane, by varying the relative motion of otoliths with the *macula sacculi*^2^*.* As previously demonstrated^50^, the different thicknesses of the otolithic membrane induce differences in mechanical resistance between the otolith and sensory epithelium.

The differences in *sulcus acusticus* and otolith ratio between *P. bogaraveo* specimens and the other congeneric species, demonstrated by the results, might be also correlated to the differences in habitat, feeding habits, and soundscape.

Despite the lack of information concerning the physiological ear response related to variations in *macula* or *sulcus* size, the sensory hair cells in *macula sacculi* are likely to be affected by changes in *sulcus* depth, shape, 3D structure (planar vs. curved), and surface.

The significant difference in relative sulcus area may be due to typical alteration in this parameter concerning differences in the mobility patterns, food, feeding behavior, and spatial niche.

Higher relative sulcus area ratios have been observed in the deepest species or those with high mobility^49^. In our study, the morphometry results concerning the sulcus did not follow those in the previous literature, displaying higher values in *P. erythrinus* and *P. acarne* compared to *P. bogaraveo*, although the latter inhabits a deeper environment than the other congeneric species.

This higher relative *sulcus acusticus* surface and the larger, curved *sulcus acusticus* of *P. acarne* and *P. erythrinus* could be correlated with higher mobility in these species (especially *P. acarne*). In *P. erythrinus,* however*,* these features might be related to its benthic lifestyle.

As demonstrated by the PCA and LDA of *sulcus acusticus* parameters, *P. erythrinus* and *P. acarne*, which share similar depths and habitats, revealed marked similarities, whereas *P. bogaraveo,* which lives in the deepest strata of the water column, displayed the most different *sulcus acusticus*. However, PCA and LDA indicated that the otolith shape in the entire *P. erythrinus sagitta* was significantly different compared to those in *P. acarne* and *P. bogaraveo.*

These features could provide a reading key for *sagitta* and *sulcus acusticus* eco-morphology in the life cycle and environmental adaptation of fish.

The connection between the otoliths and the *macula sacculi* is fundamental for transducing environmental acoustic signals and for the relative motion of fish (balance). The *sulcus acusticus* is the area of the otoliths in which this connection occurs.

### Features of the texture

Furthermore, the external textural organization^23^ changes between juveniles and adults or when environmental changes occur. The differences in the external textural organization found in juveniles and adults support those reported in the literature concerning other species^39^. Figures 3b, c and 5a–h, demonstrate that our study supported this prediction. However, *P. bogaraveo* and *P. erythrinus* juveniles, compared with other species (such as gurnards)^23^ displayed a more uniform, mineralized, external textural organization.

According to previous literature^8^, improved hearing capabilities in a species are closely related to a higher value of relative sulcus area ratio. Habitat features, such as depth, feeding strategies, mobility, trophic distribution, and ontogeny, could also influence this ratio.

Hence, it may be concluded that morphological differences in *sulcus acusticus* shape and surface among species are important for comprehending the ecomorphological and eco-functional role of *sagitta* ^2,48^.

Comparing the intra-specific differences indicated by our results with those in the literature, discussing other populations, we cannot determine whether site-differences observed in *sagitta* shape are related to genetic evolution and/or adaptative response to environment. To make this distinction it would require a specific experiment in which offspring from different populations are raised in a controlled environment.

Furthermore, the knowledge about physiology and functional morphology is insufficient to provide a clear correlation between inter-specific differences among the three congeneric *Pagellus* species and their ecological and functional features. However, differences in *sagitta* morphology and morphometry among these three *Pagellus* species may be related to differences in lifestyle, ecology, and biology since they follow the ecomorphological features of *sagittae* and species ecology described in the literature.

## Concluding remarks

This study has considered a wide range of morphometric and morphological characteristics in *Pagellus* species otoliths. Despite excellent and detailed photographs provided in previous studies^9,10^, this paper provides, to our best knowledge, the first shape analyses, using R software of *P. bogaraveo* and *P. acarne* otoliths, and the first accurate SEM analyses of *P. bogaraveo, P. acarne,* and *P. erythrinus* from the study area and other regions.

An overall image of *P. bogaraveo sagitta* and its morphometrical features was created. Due to SEM imaging, we obtained, for the first time, the most accurate image of otoliths in these species and their external textural organization. This preliminary study provides grounding for an improved understanding of the structure and eco-morphological role of the *sagitta* in the life cycle of this species. Other methodologies (e.g., X-ray diffraction, auditory sensitive measurement, CT scan) are needed to deeply investigate the physiology of the *sagitta* and its ecological adaptation to the environment. The results could aid stock identification and improve understanding of the distribution of different Mediterranean populations and their differences. Improved understanding of the phenotypic plasticity and ecomorphological role of otoliths could also serve to compare the structure, morphometry, and crystalline composition of otoliths in congeneric species of *Pagellus* from different populations, to evaluate how the sagittal structures and features change according to different environments and habitats. This approach is essential to evaluate how the morphometry and shape of different sagittal areas, such as the *sulcus acusticus*, change under different environmental pressures.

It is essential to deepen the knowledge about the later asymmetry between *sagitta* pairs in *P. erythrinus* and *P. acarne* since this may affect stock differentiation based on shape analysis between populations from different sub-areas. This feature of otolith morphometry and shape could be another response to environmental pressure, which may clarify the role of phenotypic plasticity in *sagitta* development.

## Materials and methods

A total of 44 *P. bogaraveo* otoliths (n = 24), 46 *P. acarne* otoliths (n = 24), and 71 *P. erythrinus* otoliths (n = 37) were collected from trawled specimens in the southern Tyrrhenian Sea (GSA10) between March and October 2019. Fish specimen collection was authorized by the CAMP.BIOL.19 project^52,53^. Fish otoliths were collected as part of annual research surveys, all involving lethal sampling. No experiments were conducted, nor were surgical procedures performed. No procedures caused lasting harm to sentient fish, nor were sentient fish subjected to chemical agents. The care and use of collected animals complied with animal welfare guidelines, laws, and regulations set by the Italian Government.

Before otolith extraction, each specimen was measured (TL to the nearest mm), weighed (body weight (BW) to the nearest g), and dissected to evaluate the sex and the maturity stage, according to the codes of sexual maturity in fish (MEDITS, freely available at http://archimer.ifremer.fr/doc/00002/11321/). For accurate morphometric analysis and statistical comparison of the data, specimens of each species were divided into two groups, according to the sexual maturity codes (i.e., juvenile and adult individuals).

The *sagittae* were removed from the otic capsule and cleaned of tissue using 3% H_2_O_2_ for 15 min, followed by Milli-Q water. The dry otoliths were stored inside an Eppendorf microtube.

A Leica M205C stereomicroscope with a built-in LEICA IC80 digital camera was used to collect digital images of the otolith samples (Supplementary Figures S2, S3, S4).

Each sagitta was photographed twice, once with the *sulcus acusticus* facing upwards and once with the annuli side facing up.

Before being converted into binary format for contour extraction using ImageJ 1.48p software^53^ (freely available at http://rsb.info.nih.gov/ij/), the longest axis was used to orientate the images horizontally and vertically to capture clear *sulcus acusticus* images, according to the literature^20^.

### Morphometry

According to the literature^1,20,23,32^, ImageJ was used to recorded several otolith measurements: otolith length (OL, mm), otolith width (OW, mm), otolith perimeter (OP, mm), otolith surface (OS, mm^2^), sulcus perimeter (SP, mm), sulcus surface (SS, mm^2^), sulcus length (SL, mm), cauda length (CL, mm), cauda width (CW, mm), ostium length (OSL, mm), ostium width (OSW, mm), rostrum width (RW, mm) and rostrum length (RL, mm). Afterward, other otolith shape indices were calculated: circularity (OP^2^/OS), rectangularity (OS/[OL × OW]), aspect ratio (OW/OL%), the ratio of otolith length to the total fish length (OL/TL), the percentage of the otolith surface occupied by the sulcus (SS/OS%), the percentage of the sulcus length occupied by the cauda length (CL/SL%), the percentage of the sulcus length occupied by the ostium length (OSL/SL%), the rostrum aspect ratio (RW/RL%) and percentage of the otolith length occupied by rostrum length (RL/OL%). Supplementary Figure S5 provides a schematic diagram of the measured features.

### Otolith shape analysis

**Figure 6:**
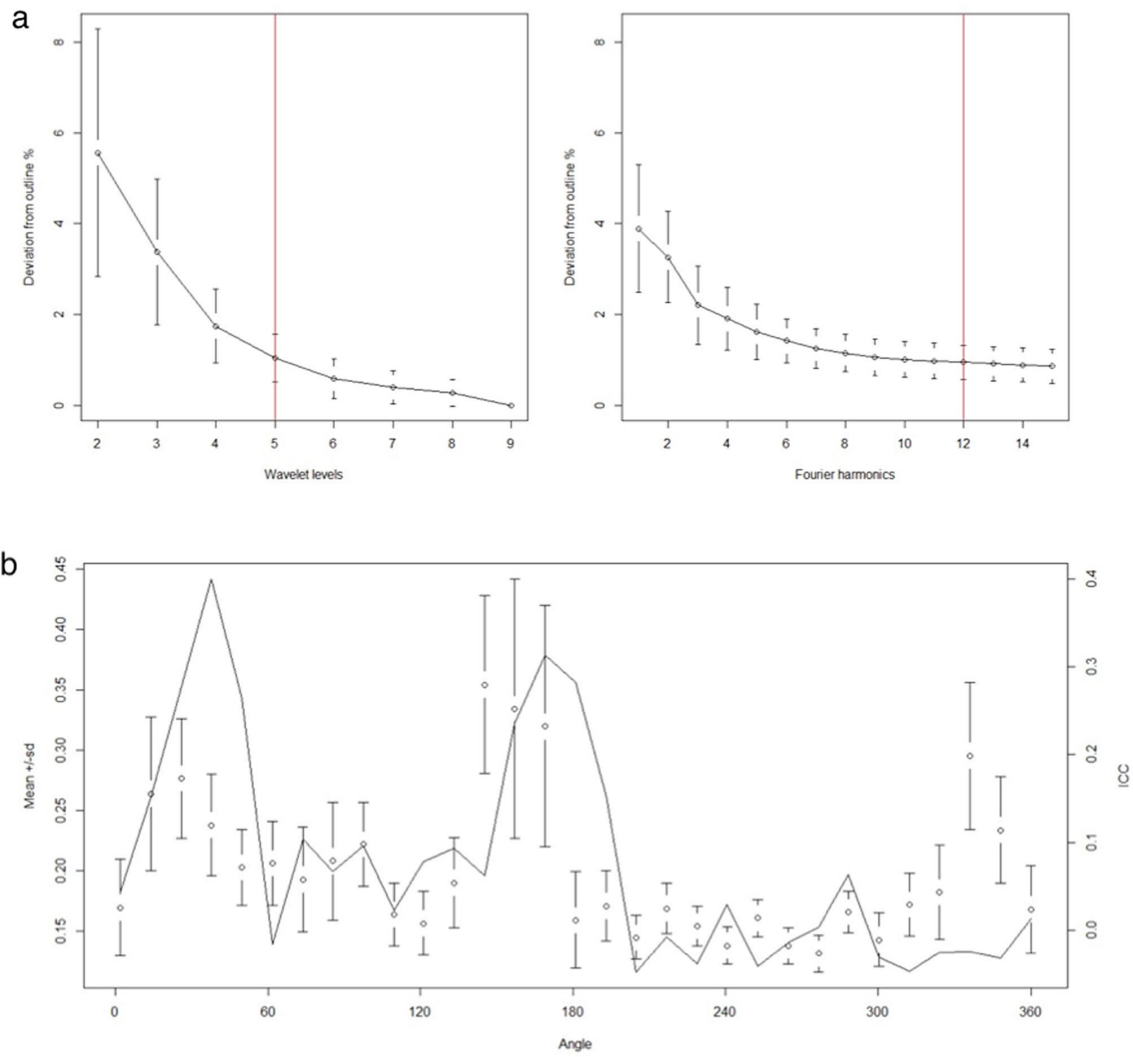
(**a**) Quality of the wavelet and Fourier outline reconstruction of left adult *sagittae* of studied species. The red vertical lines show the level of wavelet and number of Fourier harmonics needed for a 98.5% accuracy of the reconstruction. (**b**) Mean and standard deviation (SD) of the wavelet coefficients for all combined left adult *sagittae* of studied species and the proportion of variance among species or the intraclass correlation (ICC, black solid line). The horizontal axis shows an angle in degrees (°) based on the same polar coordinates of Fig. 1a, in which the centroid of the otolith is the center point of the polar coordinates.

Analysis of otolith shape was performed using shape R, an open-source software package that runs on the R platform (R version 4.0.5). This package was specifically designed to study otolith shape variation among fish populations or species^54^. First, the stereoscope captured otolith images were binarized using a threshold pixel value of 0.05 (intensity threshold). Once the outline of each image was detected, the master file containing the analyzed specimen information (e.g., fish length, weight, origin, maturity, and sex) was linked to each extracted outline. Wavelet and Fourier coefficients, required for statistical analysis, were extracted and adjusted for allometric relationships with the fish lengths. The wavelet coefficient was also used to obtain the graph shown in Fig. 1a, which compares the mean otolith shapes of the analyzed species. The quality of the wavelet and Fourier reconstruction was estimated by comparing how it deviated from the otolith outline (Fig. 6a). The maximum number of Fourier harmonics to be displayed was set at 15. Finally, the graph presented in Fig. 6b was produced by running a specific function of the g-plots R package, to investigate how the variation in wavelet coefficients is dependent on the position along the outline.

### SEM analysis

A total of four *P. bogaraveo* otoliths, three *P. acarne* otoliths*,* and four *P. erythrinus* otoliths underwent SEM analysis as described in previous studies^55^. The samples were fixed in 70% alcohol for 48 h and subsequently dehydrated using a graded series of alcohol from 70 to 100% (1 h in each solution). To avoid the critical drying point, samples were placed on a stub (SEM-PT-F-12) using conductive adhesive tables (G3347) and left for 12 h at 28 °C. Finally, the samples were sputter-coated with 20 nm gold–palladium. The samples were examined using a Zeiss EVO MA10 operating at an acceleration voltage of 20 kV.

### Data analysis

All statistical analyses were conducted using the following software: Prism V.8.2.1 (Graphpad Software Ltd., La Jolla, CA 92037, USA), R vegan package V.2.5, and PAST V. 2.7^56^.

Selected morphological parameters (OP^2^/OS, OS/[OL × OW], OL/TL, OW/OL%, SS/OS%, RW/RL%, and RL/OL%) were analysed using an unpaired *t* test to highlight any significant differences between the right and left sides of the otoliths and between juvenile and adult specimens. Differences in morphological parameters between specimens of different species (at the same maturity stage) were analyzed using a one-way analysis of variance (one-way ANOVA). Additionally, *sulcus acusticus* parameters were subjected to a principal component analysis (PCA) based on a variance–covariance matrix and Linear Discriminant Analysis (LDA) to show differences between all the analyzed species.

Finally, the correlation between the measured parameters and fish weight and total length was tested using the Pearson correlation coefficient.

To determine differences in otolith contours, wavelet coefficients were used to analyze shape variation among species using an ANOVA-like permutation test. Moreover, shape coefficients were subjected to a PCA, based on a variance–covariance matrix, and LDA to obtain an overview of the differences in otolith shape between the congeneric species examined. The significance level was set at *P* < 0.05.

## Supplementary Information

Supplementary Figure S1.

Supplementary Figure S2.

Supplementary Figure S3.

Supplementary Figure S4.

Supplementary Figure S5.

Supplementary Table S1.

Supplementary Table S2.
