# Supplementary material for: Intra- and interspecific variability among congeneric Pagellus otoliths
Source: Sci Rep. 2021 Aug 11;11:16315. doi: 10.1038/s41598-021-95814-w (PMC8357811; doi:10.1038/s41598-021-95814-w)
Supplement: Supplementary file 1 — Supplementary Figure S1. [file 41598_2021_95814_MOESM1_ESM.docx]

**Supplementary Figure S1.** Generalized a) Principal component analysis plot (PC1 versus PC2) and b) Linear Discriminant Analysis (LDA) of the *sulcus acusticus* computed between the species analysed. The PCA and LDA were based on selected *sulcus acusticus* parameters: *Sulcus acusticus* area, *sulcus aucusticus* perimeter, *sulcus acusticus* length, *ostium* area, *ostium* perimeter, *ostium* length, *ostium* width, *cauda* area, *cauda* perimiter, *cauda* length, *cauda* width, percentage of the otolith surface occupied by the sulcus (SS/OS, %), percentage of the *sulcus* length occupied by the *cauda* length (CL/SL, %), percentage of the *sulcus* length occupied by the *ostium* length (OSL/SL, %). 95% probability ellipses are shown.
