# Supplementary material for: Intra- and interspecific variability among congeneric Pagellus otoliths
Source: Sci Rep. 2021 Aug 11;11:16315. doi: 10.1038/s41598-021-95814-w (PMC8357811; doi:10.1038/s41598-021-95814-w)
Supplement: Supplementary file 2 — Supplementary Figure S2. [file 41598_2021_95814_MOESM2_ESM.docx]

**Supplementary Figure S2.** Representative stereomicroscope pictures of left Sagittal otoliths of *Pagellus acarne* examined in the study. Scale bars: 3mm.
