# Supplementary material for: Intra- and interspecific variability among congeneric Pagellus otoliths
Source: Sci Rep. 2021 Aug 11;11:16315. doi: 10.1038/s41598-021-95814-w (PMC8357811; doi:10.1038/s41598-021-95814-w)
Supplement: Supplementary file 4 — Supplementary Figure S4. [file 41598_2021_95814_MOESM4_ESM.docx]

**Supplementary Figure S4.** Representative stereomicroscope pictures of left Sagittal otoliths of *Pagellus erythrinus* examined in the study. Scale bars: 3mm.
