# Supplementary material for: Intra- and interspecific variability among congeneric Pagellus otoliths
Source: Sci Rep. 2021 Aug 11;11:16315. doi: 10.1038/s41598-021-95814-w (PMC8357811; doi:10.1038/s41598-021-95814-w)
Supplement: Supplementary file 5 — Supplementary Figure S5. [file 41598_2021_95814_MOESM5_ESM.docx]

**Supplementary Figure S5.** Generalized scheme of otolith measurement described in the text. Scale bar: 1mm.
