# Supplementary material for: Intra- and interspecific variability among congeneric Pagellus otoliths
Source: Sci Rep. 2021 Aug 11;11:16315. doi: 10.1038/s41598-021-95814-w (PMC8357811; doi:10.1038/s41598-021-95814-w)
Supplement: Supplementary file 6 — Supplementary Table S1. [file 41598_2021_95814_MOESM6_ESM.docx]

Results of t-test and ANOVA carried out on selected morphometric parameters between juvenile and adult specimens of *P. bogaraveo*, *P. erythrinus* and *P. acarne*. Significant result was set at P= 0.05. OL (otolith length), OW (otolith width), OP (otolith perimeter), OS (otolith surface), SP (sulcus perimeter), SS (sulcus surface), SL (sulcus length), SW (sulcus width), CL (cauda length), CW (cauda width), OSL, (ostium length), OSW (ostial width), RW (rostrum width), RL (rostrum length), CI (circularity), RE (rectangularity), aspect ratio (OW/OL; %), the ratio of the otolith length to the total fish length (OL/TL), percentage of the otolith surface occupied by the sulcus (SS/OS, %), percentage of the sulcus length occupied by the cauda length (CL/SL, %), percentage of the sulcus length occupied by the ostium length (OSL/SL, %), rostrum aspect ratio (RW/RL, %) and percentage of the rostrum length occupied by the otolith length (RL/OL,%). ns= not significant.

|  |  |  |  |  |  |  |  |  |  |
| --- | --- | --- | --- | --- | --- | --- | --- | --- | --- |
|  | **OP^2^/OS** | **OS/(OLxOW)** | **OW/OL %** | **OL/TL** | **SS/OS %** | **CL/SL %** | **OSL/SL %** | **RW/RL %** | **RL/OL %** |
| **Comparison between Juvenile and Adults** |  |  |  |  |  |  |  |  |  |
| ***P. bogaraveo*** | ns | ns | P=0.015 | P=0.001 | ns | ns | ns | P=0.0001 | ns |
| ***P. erythrinus*** | ns | ns | ns | ns | ns | ns | ns | ns | ns |
| **Comparison between L and R otoliths** |  |  |  |  |  |  |  |  |  |
| ***P. bogaraveo*** | ns | ns | ns | ns | ns | ns | ns | ns | ns |
| ***P. erythrinus*** | P=0.02 | P=0.02 | P=0.001 | ns | ns | ns | ns | ns | ns |
| ***P. acarne*** | ns | ns | ns | ns | ns | ns | ns | P=0.01 | ns |
| **Comparison between species** |  |  |  |  |  |  |  |  |  |
| ***P. bogaraveo vs P. erythrinus*** | P=0.008 | ns | P=0.001 | P=0.001 | P=0.001 | ns | ns | P=0.0001 | P=0.0001 |
| ***P. bogaraveo vs P. acarne*** | P=0.001 | ns | P=0.01 | ns | P=0.001 | P=0.01 | P=0.01 | P=0.0002 | P=0.0001 |
| ***P. erythrinus vs P. acarne*** | P=0.001 | ns | P=0.001 | P=0.001 | ns | ns | ns | P=0.0001 | P=0.0001 |
|  |  |  |  |  |  |  |  |  |  |
